# Medication Adherence and Liquid Level Tracking System for Healthcare Provider Feedback

**DOI:** 10.3390/s20082435

**Published:** 2020-04-24

**Authors:** Nolan Payne, Rahul Gangwani, Kira Barton, Alanson P. Sample, Stephen M. Cain, David T. Burke, Paula Anne Newman-Casey, K. Alex Shorter

**Affiliations:** 1 Department of Mechanical Engineering, University of Michigan, Ann Arbor, MI 48109, USA; nolpayne@umich.edu (N.P.); bartonkl@umich.edu (K.B.); smcain@umich.edu (S.M.C.); 2 Department of Electrical and Computer Engineering, University of Michigan, Ann Arbor, MI 48109, USA; rgangwan@umich.edu; 3 Department of Computer Science and Engineering, University of Michigan, Ann Arbor, MI 48109, USA; apsample@umich.edu; 4 Department of Human Genetics, University of Michigan School of Medicine, Ann Arbor, MI 48109, USA; dtburke@umich.edu; 5 Department of Ophthalmology and Visual Sciences, University of Michigan, Ann Arbor, MI 48105, USA; panewman@med.umich.edu

**Keywords:** adherence, glaucoma, embedded sensing, eye drop medication, internet of things, fluid level sensing

## Abstract

A common problem for healthcare providers is accurately tracking patients’ adherence to medication and providing real-time feedback on the management of their medication regimen. This is a particular problem for eye drop medications, as the current commercially available monitors focus on measuring adherence to pills, and not to eye drops. This work presents an intelligent bottle sleeve that slides onto a prescription eye drop medication bottle. The intelligent sleeve is capable of detecting eye drop use, measuring fluid level, and sending use information to a healthcare team to facilitate intervention. The electronics embedded into the sleeve measure fluid level, dropper orientation, the state of the dropper top (on/off), and rates of angular motion during an application. The sleeve was tested with ten patients (age ≥65) and successfully identified and timestamped 94% of use events. On-board processing enabled event detection and the measurement of fluid levels at a 0.4 mL resolution. These data were communicated to the healthcare team using Bluetooth and Wi-Fi in real-time, enabling rapid feedback to the subject. The healthcare team can therefore monitor a log of medication use behavior to make informed decisions on treatment or support for the patient.

## 1. Introduction

Glaucoma is the leading cause of irreversible blindness worldwide and the third leading cause of irreversible blindness in the U.S. [1,2]. Although glaucoma is initially asymptomatic, worsening glaucoma-related vision loss leads to steep declines in health-related quality of life and increased risk of falls and motor vehicle accidents [3,4,5]. Ultimately, uncontrolled glaucoma leads to blindness [6]. High rates of poor adherence to effective medications is a key modifiable barrier to better outcomes for people with glaucoma [7,8,9,10]. Interventions with patients for medication adherence have also been shown to improve use outcomes [11,12,13,14]. The National Institutes of Health Adherence Research Network has identified improving adherence to treatment and prevention regimens as a top priority” because of the promise of substantial improvements in public health as well as savings in healthcare costs” [15]. Thus, it is imperative to develop and rigorously evaluate technologies that can support interventions to improve medication adherence.

There are currently no standard methods for tracking medication adherence [16,17]; however, a variety of strategies are used including smart pill containers” [18,19,20] and wearable or ingestible sensors [21,22,23,24]. Each strategy has its strengths and limitations. Smart pill containers consist of instrumented packaging large enough to enclose the medication prescribed for the intervention. These containers use electronic systems to track the time when the user opens the top of the smart container bottle, then logs and transmits the data (typically through wi-fi or cellular services) to a database to share with the researcher or healthcare provider. These containers are simple, non-invasive, and can accommodate medication bottles in a wide range of shapes and sizes. However, the containers are designed for solid medications rather than liquid eye drops. Additionally, adherence estimates from the container can be unreliable because of the assumption that medication was taken by the patient every time the smart pill container was opened. Subject compliance significantly affects the data from smart containers. Medications taken on the go require the patient to carry the medication within a smart container. The size of this smart container results in a physical inconvenience that often results in the subjects leaving the medication behind or removing the medication from the smart container. Furthermore, these smart pill containers” are not meant to track liquid medication use. The assumption is made that liquid medication is used when removed from the larger pill container, but there is no objective evidence collected to verify its use. The ability to track adherence of liquid mediation would allow researchers and healthcare providers to more accurately assess the effectiveness of certain medications and allow adherence interventions to be applied to a wider range of medications. 

Adherence monitoring systems designed specifically for liquid medication are lacking. One of the few approaches in the literature or market place for tracking eye drop use, a very common liquid medication, was developed in Europe, where eye drops are transferred from the prescription bottle into a custom container with sensors to quantify use (Nemera, La Verpillière, France). These sensors include an inertial measurement unit (IMU) for position and shaking detection, Global Positioning System (GPS) for location tracking, and wireless capabilities to communicate with a smartphone. However, this approach requires the use of the company’s own custom eye drop medication bottles to latch to a system that contains the embedded sensors. As a result, this product is not currently approved for use in the United States because this system alters the dispensation of the medication. To receive approval, the Nemera system would require a full set of Food and Drug Administration (FDA) drug trials. To mitigate the extensive time and costs associated with FDA drug trials, a monitoring system capable of detecting and quantifying eye drop medication use without altering the original medication packaging must be developed. 

To address this gap, this work describes the design and fabrication of a portable sensor system that surrounds an approved eye drop medication package for the adherence monitoring of liquid medication. The sensors reliably detect medication use, measure the level of the fluid-based medication, and transmit that information to the healthcare team rapidly. The ability to accurately monitor medication frequency of use and the amount of medication in the package will give clinicians important information about patient adherence in real-time, enabling the opportunity to provide timely feedback to improve adherence.

## 2. Materials and Methods

### 2.1. Overview

**Figure 1:**
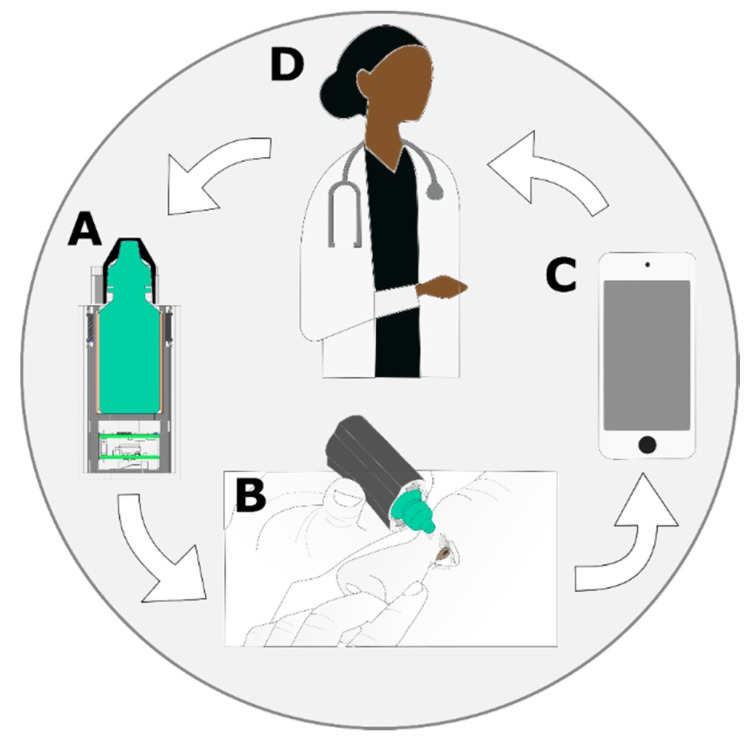
Overview of information flow in the system. (**A**) The prescription bottle is placed in the sleeve with the embedded sensors and electronics. (**B**) Data from the sensors detect use and monitor fluid level. (**C**) Data and usage information are transmitted from the system to a smart phone or another Bluetooth connected device. (**D**) Healthcare providers use this information to inform clinical decisions.

This work used an integrated approach, combining embedded sensors, real-time state classification, and communication to and from the embedded system to quantify adherence and fluid level for glaucoma medication (Figure 1). A custom embedded system was designed, fabricated, and tested, both in the laboratory and with subjects, in a controlled environment to provide sensor information. The system does not modify the existing eye-drop medication bottle in any way. The on-board processor runs real-time classification algorithms to detect and log each use event. These events as well as fluid level are transmitted to a Bluetooth enabled device. Then, the information is communicated to the healthcare provider through Wi-Fi. The logged information can be used to provide feedback to the patient with visual alerts from LEDs on the sleeve or digital reminders that can be set up to notify the patient when the medication needs to be taken or refilled. 

### 2.2. Sleeve Design

#### 2.2.1. Sleeve Packaging and Electronics

**Figure 2:**
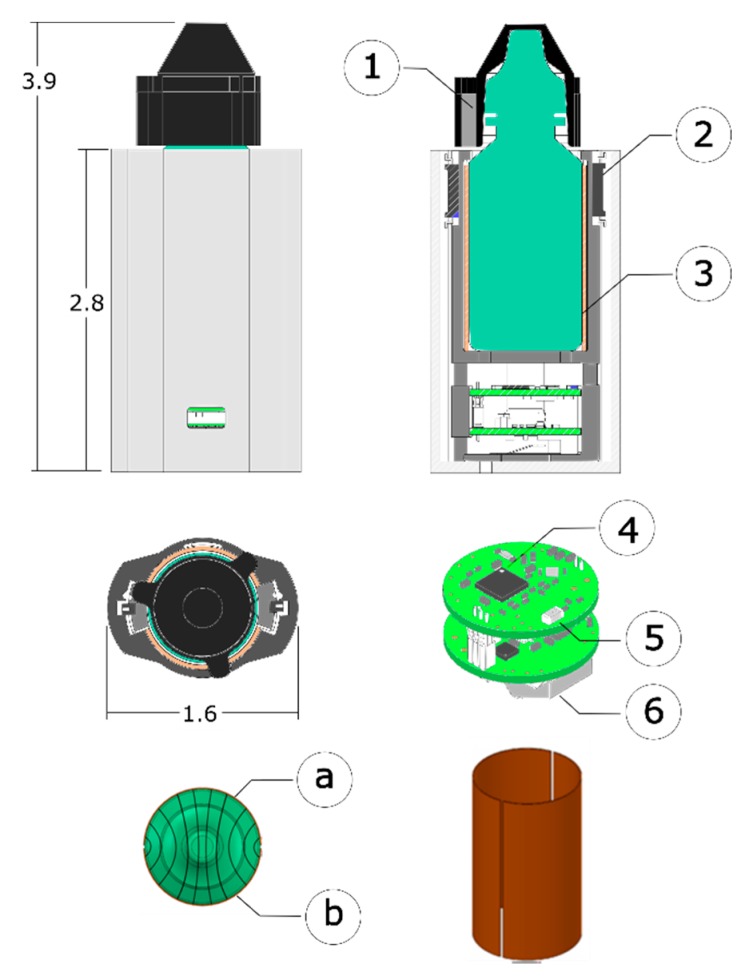
Mechanical layout of the bottle and sleeve assembly. (**1**) Bar magnets placed in the cap, and (**2**) reed switches in the sleeve are used to sense the cap removal. Electronics are embedded in the base of the system and were designed around an (**4**) nRF51422 system-on-a-chip. (**5**) BLE was used to transfer data, and the system was powered using (**6**) a single rechargeable coin cell battery. (**3**) The two-part capacitive sensor consisted of two rectangular copper sheets (**a**) and (**b**) surrounding the bottle. The bottom left view illustrates the electric field measured by the capacitance sensor.

The sleeve was designed as a low power, portable Internet of Things (IoT) system for day-scale use (Figure 2). The low power nRF51422 system-on-a-chip (SoC) was selected as the main processing unit and Bluetooth low energy (BLE) was selected for data communication [25]. BLE was chosen over high speed data transmission to further decrease the power consumption [26]. BLE uses a Gaussian frequency shift keying (GFSK) modulation, which allows reduced peak power consumption. Unlike Bluetooth, BLE uses two types of RF channels, advertising and data transmission, to poll for a connection and send data at set intervals [27]. BLE takes advantage of duty cycling data transmission, which when combined with the nRF51422 processor’s ability to switch between an active and sleep mode, enables efficient active and idle modes of operation (Table 1).

To allow for easy size adjustments for different bottle shapes and sizes, the mechanical components of the sleeve were 3D printed. The sleeve (Figure 2) consists of three components: (1) the bottle cap cover with magnets, (2) a structural ‘skeleton’ used to co-locate the electronics and sensors, and (3) an elastomeric sleeve that encases the system and facilitates eyedropper use by the patient. The ‘skeleton’ and ‘sleeve’ were fabricated using Formlabs Form 2 printers and the cap cover was fabricated with an Ultimaker 3 Extended printer from thermoplastic polyurethane (TPU) filament.

Two reed switches and magnets embedded in the 3D printed cap detected the state of the bottle top. An inertial measurement unit (IMU, BNO080) with a 3-axis accelerometer, gyroscope, and magnetometer provided information about the bottle orientation [28]. To complement the orientation sensing, a custom capacitance sensor was designed to measure the fluid level without modifying the prescription bottle. The sensor consisted of two plates” made from copper tape formed into a cylinder, as illustrated in Figure 2. These plates act as two capacitors in parallel, with the top capacitor measuring the empty volume of the bottle (filled with air) and the bottom capacitor measuring the medication. The total capacitance of the air/fluid mixture in the bottle is the sum of the capacitance. Therefore, with known dielectric constants of the air and fluid and a baseline capacitance reading across the full bottle, the fluid level can be calculated as follows:

(1) $$\;C_{total}=C_{Medication}+C_{Air}\;$$

(2) $$\;C_{total}=R*V+C_{empty}\;$$

where R represents the ratio of the dielectric constants of medication to air multiplied by the geometric constant related to the shape of the bottle; V represents the volume of medication remaining in the bottle; and $C_{empty}$ represents the capacitance reading when no medication remains in the bottle. The cylindrical sensor attaches on the inside of a thin insulating sleeve made from a 3D printed silicone-like flexible material (Formlabs Elastic, USA). A capacitance to digital converter (FDC1004) measures the capacitance across the volume of the medication bottle.

Two printed circuit boards (PCBs), each with the same area as the footprint of the bottle, were fabricated for the electronics (Figure 2). The top board contains the microcontroller, nRF51422 chip, and an impedance matching network to maximize the efficiency of the antenna. The bottom board contained the IMU, capacitive sensor, battery holder, headers for wires to the reed switches, and an MCP73831 IC to charge the lithium-ion coin cell batteries. Two LEDs provided visual indication of the bottle power and bottle state”: In state (1), the bottle is polling for a BLE connection (flashing LED), or state (2), a connection has been established between the bottle and a peripheral Bluetooth device (LED off). A 3.7 V (85 mAh) lithium-ion battery (RJD2032C1) provided power to the system. 

#### 2.2.2. Data Logging and Communication

The sleeve works in tandem with a commercially available Bluetooth-enabled device for data storage and communication of events and measurements. The sleeve system operates in the following manner: (1) power on, (2) wait in an idle state, polling for a BLE connection every 40 milliseconds until 30 s have passed, and (3) time out and wait 30 s before trying again. When the reed switches data readings indicate that the cap is off, an internal timer is activated and the processor polls for sensor data. The sensor data are used to determine if a use event has occurred. Additionally, the system queries the IMU every 30 min to determine if the bottle is stationary and upright, in which case, the fluid level is measured using the capacitive sensor. When the sleeve is connected to the Bluetooth enabled device, data are sent via BLE to a custom application on the communication device that logs and timestamps the received data. For the initial testing, an iOS-specific application was developed in Swift 3.0 that both receives and redirects data to a web server. The application was developed on top of the Core Bluetooth Module library, which allows for easy auto connect in background mode, freeing the user from direct interaction with the app. If the bottle is not connected to the peripheral device, all data are stored in the internal memory until a connection can be made.

### 2.3. Event Detection

#### 2.3.1. Event Detection – Rule-Based Algorithm

An offline rule-based algorithm was used to verify that features from the sensor data could be used to determine use events. The three chosen rules for the offline event detection algorithm were: (1) the cap state was off, (2) the orientation (based off the IMU data) was between 100° and 260° with respect to the vertical axis along the height of the bottle, and (3) there was a positive increase in the capacitance data for at least four consecutive data points (signifying force application).

#### 2.3.2. Event Detection – Machine Learning Algorithm

For the system to be effective, it is crucial to reliably determine when medication has been used by the patient online. To identify a use event automatically, sensor data were used to identify and train a classification algorithm to run, in real-time, on the microprocessor. Sensor data gathered when the sleeve assembly was dispensing liquid were then used to create features using descriptive statistics (see Appendix B for featurization approach), and labeled according to the usage events (i.e., lid off with both an orientation and capacitance change). Weka [29] was used to train and compare the classification performance of different supervised machine learning algorithms. The information gain attribute in Weka identified the worth of features extracted from the sensor data by measuring the information gain with respect to the class [30]. The information gained by each sensor was then found by averaging the information gained by each feature relative to the sensor. The skill scores of the models were verified using k-fold cross validation, where 90% of the dataset was trained by k-1 folds and 10% was tested on the last fold [31]. The random forest algorithm, which uses a combination of trees and averaging to determine the optimal threshold for the features with the highest identified information gain, was selected for use with the system.

### 2.4. Experimental Evaluation 

#### 2.4.1. System and Sensor Testing

To determine the fluid level sensor resolution, two sets of tests were performed. First, a full-range bottle test determined the capacitance changes as the volume of the fluid was removed in 1 mL increments from the bottle. Second, the resolution and repeatability of the readings were evaluated over a reduced volume range. Both tests used a 15 mL bottle of lubricant eye drops and sampled the capacitance sensor at 10 Hz. For the full level test, the bottle was filled to 15 mL, and a micropipette removed fluid in 1 mL increments until the bottle was empty. To test sensor resolution and repeatability, the bottle was filled with 8 mL of liquid and a total of 2 mL was removed in 0.2 mL increments. For both tests, the mass of the bottle was recorded from a 1 mg resolution scale to verify the remaining fluid level. The capacitance readings from the FDC1004 sensor chip were logged to match the bottle volume to the steady-state capacitance value. The bottle was kept in an upright position for the readings. Each test was repeated five times. Linear regressions were calculated to characterize volume versus capacitance data trends.

To determine the range of BLE, an iPod Touch communicating through BLE was placed in one corner of a room. A fully assembled sleeve, placed near the iPod Touch, was enabled with BLE and was constantly pinging data to the iPod Touch. The sleeve was then moved away from the receiver until the iPod Touch no longer received data from the sleeve. A tape measure was used to measure the distance between the iPod Touch and the sleeve. This test was repeated five times, and the results were averaged to provide a metric describing the range of BLE communication.

To quantify the power consumption of each component of the PCB, an ammeter was used at test points around the PCB to measure the current draw for each sensor. Power consumption was determined for the following bottle states: (1) polling for a BLE connection, (2) in ‘idle’ state waiting for cap removal, and (3) in ‘active’ state running all sensor components. The measured power consumption was used to estimate the time until recharging was required. 

#### 2.4.2. Experimental Evaluation – Subject Testing

To test the bottle electronics and detection algorithms, ten participants aged 65 or above were recruited to perform a series of tasks in a controlled experimental environment. All protocols were approved by the Institutional Review Board at the University of Michigan (IRB # HUM00162357). After the study coordinator obtained written informed consent, each participant received a bottle and performed specific tasks to simulate how the bottle might be used in a real-world scenario. A Wi-Fi enabled iPod Touch was placed in the center of the room to collect and store data transmitted from the system, and video of the participants was collected as they performed six tasks:

1. Walk around the room with the instrumented system in a pocket/purse for one minute.
2. Remove the bottle cap, dispense eye drops (in one or both eyes depending on personal preference), then place the cap back on the bottle. Repeat five times.
3. Remove the bottle cap, place the bottle back on the table without dispensing any fluid, then place the cap back on the bottle. Repeat five times.
4. Shake the bottle for five seconds (with the cap still on). Repeat five times.
5. Remove the bottle cap, simulate the motion used to dispense eye drops, but do not dispense fluid, then place the cap back on the bottle. Repeat five times.
6. Remove the bottle cap, dispense eye drops in the same fashion as Step 3, but with the participant reclining, then place the cap back on the bottle. Repeat five times.

The ten subjects each used the eyedropper ten times (fluid was dispensed five times each in tasks 2 and 4), resulting in 100 use events. Additionally, the cap was also removed from the bottle without dispensing medication a total of 100 times (tasks 3 and 5). Data were synchronized with the video recordings and labeled by a trained observer. The labeled data were used to train the detection algorithms and evaluate how features from the sensors contributed to correct event detection (as described in Section 2.3). The labeled data were used with both online and offline algorithms to identify use events.

#### 2.4.3. Experimental Evaluation – Full-Day Testing

Pilot testing of the on-board detection algorithm, built from the experimental data described above, were tested during day scale use tests with a single subject. The bottle was set to run for the entire day, and each hour, the participant dispensed medication. During the trial, the participant carried the instrumented bottle with medication over the course of a regular work day (~7 h), dispensed medication hourly, and logged a time stamp of use for comparison with the algorithm’s results. The eyedropper was programmed to send capacitance data every 30 min when the bottle maintained an upright position, which was verified through the accelerometer. An iPod Touch received data from the sleeve, and the participant came into range of the receiver every three hours. At the end of the day, data from the iPod Touch were exported to an Excel Spreadsheet, where the results from the on-board detection algorithm were compared to the participants’ log. 

### 2.5. Statistical Analysis

A non-parametric test for each pairwise comparison of the experimental volume levels was used for the experimental characterization of the capacitance sensor. The Mann–Whitney U-test was selected because it is appropriate for small sample sizes (five replicated experiments). Multiple comparisons were not used as each pairwise-comparison was assessed separately. The range of comparison *p*-values, rather than each of the individual comparisons, were reported. 

Precision, Recall (true positive rate), and F-measure (F1 Score) metrics were used to compare the multiple machine learning algorithms and machine learning with the rule-based algorithm. These metrics are calculated as follows:

(3) $$\;Precision=\frac{TP}{TP+FP}\;$$

(4) $$\;Recall\;\left(TPR\right)=\frac{TP}{TP+FP}\;$$

(5) $$\;Fmeasure\;\left(F1\;Score\right)=2*\frac{Precision*Recall}{Precision+Recall}\;$$

where TP is the True Positive and FP is the False Positive.

## 3. Results

### 3.1. System and Sensor Testing

**Figure 3:**
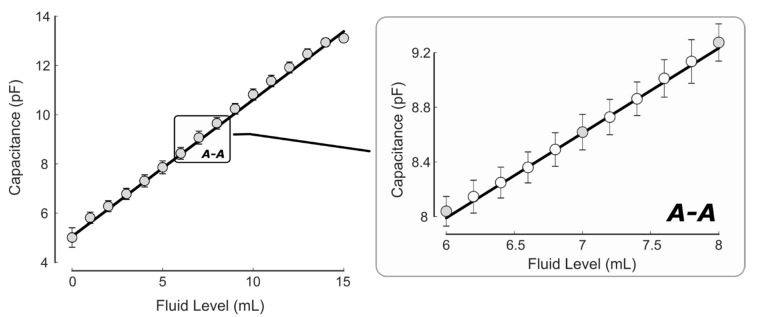
Fluid level sensor calibration results for changes over the entire bottle volume (**left**) and a higher resolution test with 0.2 mL increments (**right**). Results indicate a linear relationship between fluid volume and capacitance reading with a resolution of approximately 0.4 mL.

The results of the fluid level testing of the capacitive sensor are presented in Figure 3. The capacitance changed linearly with fluid level, with small variability between the trials. The linear regression for the full level test data resulted in the following relationship:

(6)  C=0.56∗V+5.15 

with *C* representing the capacitance reading and *V* representing the fluid volume. The regression fit the data well with an R^2^ value of 0.998. Results from the high-resolution fluid test (Figure 3) indicated that a 0.2 mL change in fluid level was not statistically different (ten pairwise comparisons, *p*-value range = 0.174 to 0.23). However, fluid changes of 0.4 mL resulted in statistically significant differences (nine pairwise comparisons, *p*-value range = 0.012 to 0.037). The error bars on the graph represent the sample standard deviation for the five repetitions at each fluid level. 

The results of the range test indicate that, on average, BLE was able to communicate up to 30 feet before the connection between the instrumented bottle and iPod Touch was lost. Table 2 provides a power budget analysis for two modes of operation. These results consider the system use with the online machine learning algorithm for event detection. Given the chosen battery, the calculated lifespan of a single charge was about 17.5 h (see Appendix A for details). 

### 3.2. Patient Testing

**Figure 4:**
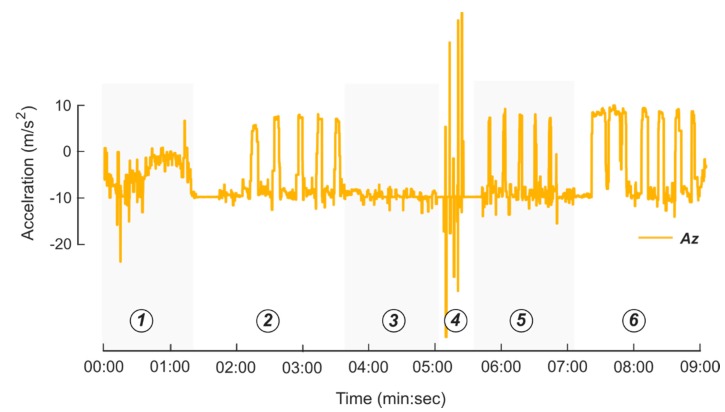
Example Institutional Review Board (IRB) trial data for *z*-axis accelerometer from one participant. (**1**) Participant walked with the system in a pocket/purse for one minute. (**2**) Participant dispensed medication five times while in a standing position and placed the eyedropper on the table between each use. (**3**) Participant removed the cap from the eye dropper and placed the eye dropper on the table without dispensing medication five times. (**4**) Participant shook the sleeve with the cap still on five times. (**5**) Participant removed eye dropper cap and executed a simulated eye drop event five times. (**6**) Participant dispensed medication five times while in a reclined position and placed the eyedropper on the table between each use.

**Figure 5:**
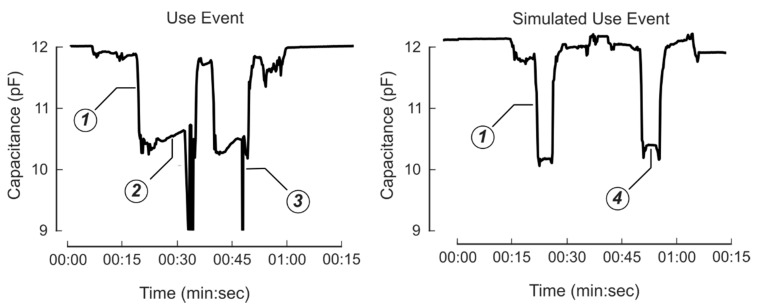
Raw capacitance data of a use event (**left**) and a simulated use (**right**). For both cases, the capacitance dropped as the bottle was flipped (**1**) because the fluid flowed out of the bottle body and into the nozzle. There was a gradual increase in capacitance for the use case (**2**), which was caused by the participant squeezing the bottle. Then, there was an occasional sharp drop caused by the suction of air after the participant finished the squeezing action (**3**). Neither the slope nor the spike was present in the simulated use case (**4**).

**Figure 6:**
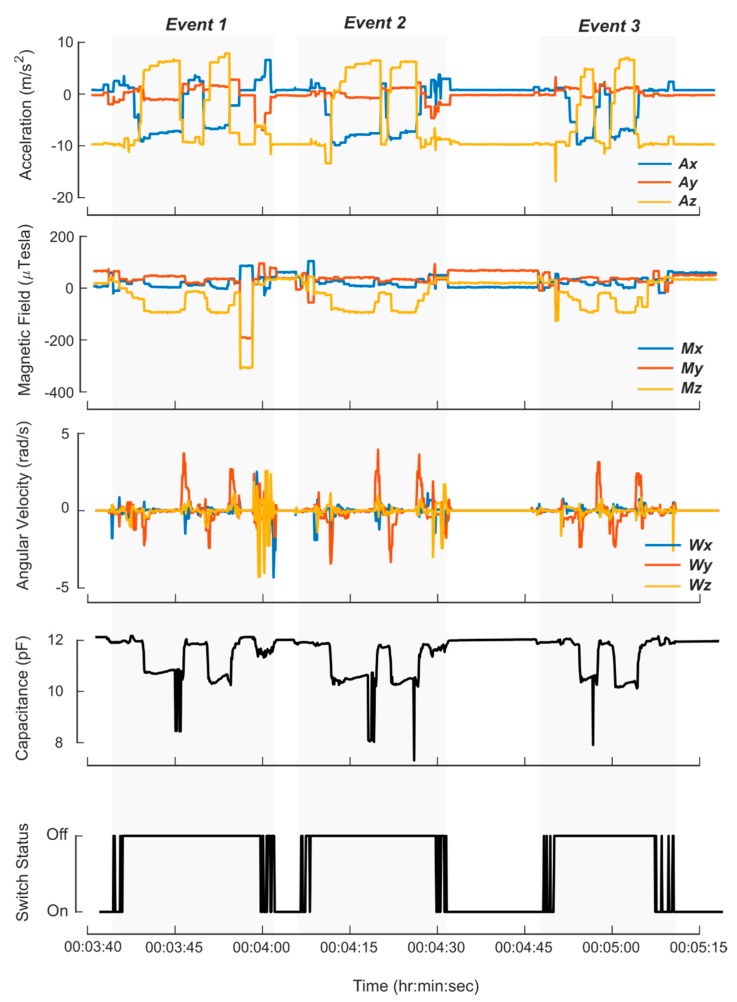
Data from the sleeve during three consecutive use events. Reed switch status indicates cap state (on or off). When the bottle was inverted, there was a change in orientation most clearly visible in the accelerometer data. The changing angular velocity at the beginning and end of each application was present in the recorded angular velocity. Data from the capacitance sensor showed the initial drop in capacitance was due to movement of the fluid into the top of the bottle, and then the additional drop in was capacitance created as the droplets were dispensed.

Data from each sensor were successfully logged for the ten participants. Features from the sensor measurements demonstrated clear differences between the six tasks performed in the protocol. The *z*-axis acceleration from a representative subject trial is presented in Figure 4. The IMU was oriented with the *z*-axis vertically along the height of the sleeve in the downward direction. For example, when the bottle was in the upright position, the *z*-axis acceleration was approximately 9.8 m/s^2^. A trained observer reviewed the video and classified the six tasks. There were clear differences between the ‘everyday actions’ such as walking or shaking, and the use events. Sensor data collected during the simulated eye drop use events (no fluid dispensed) showed similar features compared to the real use events, but there were clear differences in the capacitance data. Figure 5 shows an example of the capacitive measurements of an actual use event for both eyes on the left, and a simulated dropper use event for both eyes on the right. When the bottle was inverted, the capacitance reading decreased as fluid moved into the top of the bottle. For the actual use case on the left, the participant applied a force to the bottle to dispense a droplet, resulting in an increased reduction in capacitance. This feature was not present in the capacitance measurement during the simulated trial. Figure 6 shows the sensor data from three pairs of use events. When the bottle was flipped in order to dispense liquid, the *x*-axis and *z*-axis accelerometer data flipped and there were minor spikes in the gyroscope data.

### 3.3. Machine Learning Results

**Figure 7:**
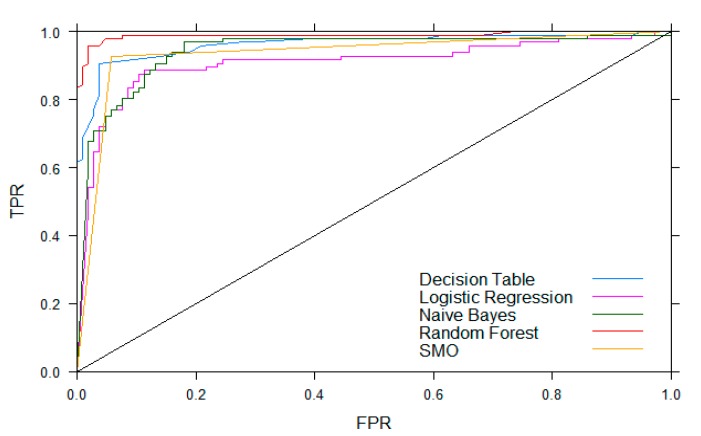
Receiver operating characteristic (ROC) curves of each algorithm. ROC curves plot the false positive rate (FPR) versus the true positive rate (TPR). Therefore, the closer the ROC curve is to the upper left corner, the higher the overall accuracy of the model.

**Figure 8:**
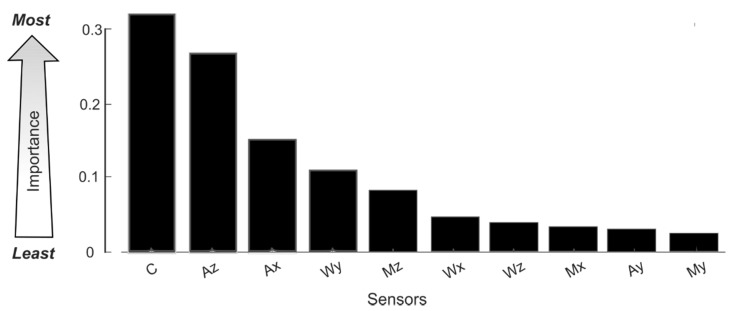
Information gained (IG) is the percentage of total information the model gained by a feature in classifying use events. Using the average IG of all features with respect to the sensor, the importance of a sensor in classifying a use event can be determined. Sensors ranked were the accelerometer (A), magnetometer (M), gyroscope (W), and capacitance sensor (C). Data from sensors related to the orientation of the bottle and the capacitance were the most important. Reed switches were not included in importance rankings because the built in algorithm only runs when the reed switches indicate the cap has been removed.

The comparison of machine learning algorithms when classifying use events is shown in Table 3, and receiver operating characteristic (ROC) curves for each algorithm are shown in Figure 7. Based on the true positive rate (TPR), false positive rate (FPR), and area under the ROC curves, we determined that the best algorithm to detect use was the random forest classifier. Weka includes a method for evaluating the importance of features with its information gain attribute. Appendix B includes a table that presents the total percent of information gained by each feature. Figure 8 shows the average information gained with respect to each sensor using the table in Appendix B. Features from sensors that provide information about the orientation of the bottle and the capacitance sensor ranked as the most important.

### 3.4. Machine Learning versus Event Detection Algorithm Results

Offline and online use event algorithms were built from the subject trial data. The online event detection algorithm parsed data into windows based on the status of the cap, created features from the sensor data, and passed those data into a machine learning procedure, while the offline event algorithm was built in MATLAB by observing and testing different rule” cut-offs with the trial data. The random forest (online) and rule-based (offline) algorithms were evaluated using the subject trial data as the testing set and the results are provided in Table 4.

The online random forest algorithm had a true positive rate (TPR) of 97% compared to 92% from the offline algorithm. The online algorithm also had a false positive rate (FPR) of 11% compared to 23% from the offline algorithm. The false positives were from the simulated use events flagged as real use events of the medication. The false positives were also from incorrectly flagged cap removals without medication dispensing events. These incorrectly identified events occurred when a participant squeezed the bottle to remove the cap with the eyedropper nearly horizontal. These events were flagged as use events because they executed behavior similar to the start of a use event when the participant begins moving the medication toward their eye. The cap removal detection used with both algorithms was accurate and filtered out all events that did not require cap removal. 

### 3.5. Full Day Test Results

With the machine learning algorithm running on the eye dropper’s printed circuit board (PCB), the instrumented eye dropper classified all usage events from the day scale trials correctly. Out of the seven times that the researcher dispensed medication, the bottle successfully recognized that the cap was off, ran the machine learning algorithm, and timestamped seven use events. The reed switches reliably detected the cap state, and the features and thresholds determined for the classifier were accurate enough to classify a use event at 100% accuracy, given proper use by a lab researcher. The researcher also observed every time a capacitance reading was recorded. When the bottle was standing still at an upright position, capacitance readings were recorded every 30 min. During one 30-min period when the researcher traveled with the bottle, the capacitance was not recorded. Fluid level measurements were only made when the bottle was in an upright position. 

## 4. Discussion

Embedded sensing and electronics have the potential to greatly improve medication adherence and the consistency of medical interventions. Most healthcare providers currently do not have real-time information about when or how much medication their patients are using. Devices like glucose monitors or digital pedometers can provide meaningful feedback to patients. However, integrated systems that pass health monitoring data to or from both patients and clinicians in real-time are lacking. Additionally, systems to monitor medication adherence in real-time are currently only used in research because of their high cost and cumbersome design. Embedded systems to measure eye drop use, a particularly unique type of medication, and transmit the information both to patients and providers has great potential to improve medication adherence and outcomes. Patient access to information about when and how much medication they are using could motivate higher levels of medication adherence. Information from the bottle and supplementary reminders from the clinicians could also be used to reduce the amount of time that the patient is without medication. 

In addition to providing feedback to an individual patient, the proposed system for monitoring glaucoma medication has the potential to facilitate research that is necessary to quantify and improve patient adherence. Clinical studies to investigate the impact of improving adherence on biological outcomes require hundreds of patients. Current state-of-the-art systems can cost as much as $200 per month per medication for monitoring fees (personal communication, AdhereTech, NY, NY). For a study of 1000 subjects over the course of a year, this would result in an estimated cost of over $2,000,000. For this preliminary study, we made ten instruments at a total cost of ~$500, and we estimate that monthly monitoring fees will accrue at a rate of approximately $10/month for small data use plans. Additionally, the price per unit will drop when hundreds or thousands of sleeve systems are fabricated, creating a price point that would likely reduce the financial barriers that currently limit access to adherence monitoring. A lower price point would facilitate both large clinical studies and potentially ubiquitous medication monitoring for all patients with glaucoma, or at least for those in whom adherence is poor and/or the disease is worsening.

For glaucoma medication, in particular, creating a system to augment existing FDA approved medicine bottles with embedded electronics is a necessary design feature, and there are currently no devices in the literature or market place of this type. The system presented here integrates sensors to monitor use and measure fluid level in a streamlined package, capable of real-time use classification and fluid level measurement. This information can also be shared in real-time and does not require patient input other than taking their medication as they normally would. 

To assess the accuracy and reliability of the system, a series of trials with the instrumented bottle were conducted with subject participants 65 years of age and over. For the online detection, a random forest algorithm was chosen over other optimal classification algorithms based on the results of cross validation. Random forest uses a combination of trees and averaging to determine the optimal threshold for features to achieve the highest accuracy [31]. The machine learning algorithm (online) for detecting use implemented with the system was able to successfully identify 97% of the use events. During testing, three out of 100 use events were not correctly classified. During these misclassifications, the magnitude and rate of the capacitance sensor data were lower and slower when compared to the correctly classified use cases, possibly indicating a slower application of the medication. The online algorithm did have false positives (11%). Most false positive detections occurred when the participants performed a ‘simulated’ use, attempting to trick” the system by raising the bottle to their eye without applying force to release fluid. This limitation can be partially mitigated by using the fluid level sensor in combination with use detection to provide context about how medication level is correlated with state classification. In terms of fluid measurements, the bench test results showed that the capacitance sensor was able to measure fluid in the bottle at a 0.4 mL resolution. This means that while the fluid level sensor is not yet capable of detecting fluid level change on a single drop basis, it is capable of measurements in the five to ten drop range. During the day-long medication use trial, where fluid was dispensed each hour, the online algorithm was able to successfully identify all use events without any false readings.

The online random forest algorithm also outperformed the offline rule-based method investigated in this work. This can be seen by comparing the F1 score, which is the weighted average of the Precision and Recall values. Since F1 considers both Precision and Recall, a higher F1 score means that the model has lower false positives and false negatives if the Precision and Recall values are also both higher. The online algorithm obtained an F1 score of 0.93 and the offline obtained a score of 0.86, so the online algorithm had less false positives and false negatives. The machine learning algorithm was able to out-perform the rule-based algorithm for a couple of reasons. First, the machine learning algorithm looked at the summation of the absolute value of all changes of data in order to capture force variations due to the capacitance sensor. By looking at the absolute value, the algorithm captured the minor spikes in data that occurred when the user squeezed the bottle to dispense fluid (see Figure 5). Second, the rule-based approach used observations of MATLAB graphs to determine the appropriate thresholds to classify a use event. In contrast, the machine learning approach used a well-defined algorithm to observe all training data in order to generate the appropriate thresholds.

To qualitatively assess the streamlined packaging, the researchers asked the participants if they had any difficulties or concerns with the sleeve. Three of the ten participants mentioned that the sleeve made the eyedropper more difficult to squeeze. After reviewing the video recordings from these participants, it was clear that this difficulty was a result of hand placement near the bottom of the eye dropper. The sleeve added length to the bottle, resulting in hand placement lower than what the subject might normally expect. More time to familiarize themselves with the sleeve and the addition of colored marks or features to indicate correct hand placement would address this observation. Three participants also mentioned that the cap cover made the top of the bottle easier to remove. The cover made the cap larger, and the magnets allowed for an easier location to grip and twist off the cap.

Currently our system can monitor adherence for ~17 h, enough time to operate during the day on a single charge. To improve power efficiency of the system going forward, a few changes can be made. First, the sensors that were chosen require more power than necessary. As can be seen from the results of the information gain evaluation, the implemented on-board detection algorithm could accurately classify use events without using features from the on-board gyroscope or magnetometer. Removing the gyroscope and magnetometer as well as selecting more power-efficient accelerometers would improve the system power consumption. Second, as seen in the power budget, the majority of power consumption was due to BLE. Since the purpose of this version of the system was to reliably communicate sensor data with a web server, BLE was optimized with the maximum settings in order to establish a steady connection. However, the trade-off of reliable communication is an increase in power consumption, which can be avoided by changing parameters such as the advertising and connection intervals. Since the bottle spends the majority of its time polling for a BLE connection, by reducing the advertising interval, the overall power consumption will decrease [26]. By decreasing power consumption, the bottle can monitor adherence for a longer period of time.

Improved manufacturability of future devices will be key to the creation of a low-cost system for persistent monitoring. For example, the reed switches were reliable and convenient, but require manual assembly because they need to be soldered to wires, glued into place, and wired down into the PCB. A different sensor such as a dedicated magnetometer could do the same job and would improve the manufacturability of the system. Finally, 3D printing of the sleeve is convenient because it can be easily adjusted for different sized bottles; however, 3D printing makes the manufacturing more expensive and limits the materials that can be used. Other manufacturing methods such as injection molding can be used to manufacture the larger parts such as the silicone-like outer sleeve, with 3D printing used to make inserts that adjust to different sizes.

## 5. Conclusions

Accurately tracking patients’ adherence to medication is an issue that has not been well addressed, especially when it comes to eye drop based medications. Utilizing a select group of off-the-shelf and custom-built sensors combined with a Bluetooth enabled device, we developed a sensor system capable of detecting eye drop use, measuring fluid level, and sending use information to a healthcare team to facilitate intervention. A 3D printed sleeve that fits around a prescription eye dropper enables customization for various bottle shapes and sizes. Subject testing with ten patients aged ≥65 demonstrated that the system could successfully identify and timestamp 97% of use events and was capable of measuring fluid level at a 0.4 mL resolution. 

While developing this application, we found other uses for the sensors, specifically the capacitance sensor. Though eye drops are a very common liquid medication, accounting for 2.3% of Medicare Part B expenditures or $2.4 billion annually [9], there are other liquid medications whose consumption could also be better monitored through this new sensor system compared to a standard smart pill bottle.” For example, the insulin required to control diabetes is a liquid medication. Diabetes affects 10.5% of the U.S. population, or 34.2 million people, and is the seventh leading cause of death in the USA (CDC). Insulin is most frequently dispensed in a concentration of 100 units/mL, where a dose is anywhere between 2 and 100 units of insulin, requiring a 0.3 mL syringe to measure out insulin doses in up to 0.02 mL aliquots. A future direction for this research would be to refine the ability to detect how much liquid medication is dispensed to the 0.01 mL level. This would enable not only the detection of whether or not a single drop of liquid eye medication was dispensed, but it would also enable the detection of whether a single unit of insulin was dispensed. This level of accuracy would enable health care providers to know whether both of these important types of liquid medications were being accurately taken by patients to best control their disease. 

The portable sensor system developed is not a simple, one-off device with the sole purpose of monitoring eye-drop use. Rather, the core hardware and communication system can incorporate additional sensors to capture data on individual patient environments and activities. Future designs of this system can evaluate adding or replacing sensors that will enable physicians or researchers to understand the environment and movements of the patient in more detail. This can help provide a richer understanding of the patient environment and behavior, and can tailor the adherence process to each individual patient. 

## Figures and Tables

**Table 1:** Sensors and components embedded in the sleeve, along with the model number used in the prototype and the functionality of the component.

Component	Manufacturer	Model	Function
Inertial Movement Unit	Hillcrest Labs	BNO080	Orientation Estimation
Reed Switch x2	Coto Technology	CT10-1540-G1	Cap Removal Detection
Capacitance-to-Digital Converter	Texas Instruments	FDC1004	Fluid Level ^1^ and Force Detection
Microcontroller	Nordic Semiconductor	nRF51422	Data Processing/BLE communication
Coin Cell Battery	Illinois Capacitor	RJD2032C1	Powering
Charge Management Controller	Microchip Technology	MCP73831	Charging

^1^ Combined with custom-built capacitive sensor.

**Table 2:** Power budget calculations for printed circuit boards (PCB) components after machine learning results.

Component	Idle Power (mW)	Active Power (mW)	Time Active (%)	Average Power (mW)
nRF51422 (MCU)	3.3	30.5	0.1	3.3
BNO080 (IMU)	5.0	23.5	0.4	5.1
FDC1004 (Capacitance)	0.1	2.3	0.4	0.1
Bluetooth (BLE)	8.9	32.4	3.0	9.5

**Table 3:** Comparisons of machine learning (ML) algorithms using the true positive rate (TPR), false positive rate (FPR), Precision, Matthews correlation coefficient (MCC), and receiver operating characteristic (ROC) area. All algorithms found the same number of false positives as false negatives, so the TPR (also known as Recall) and Precision values were the same. Therefore, the algorithm with the highest number of TPR/Precision and lowest FPR was the best algorithm.

ML Models	TPR	FPR	Precision	MCC	ROC Area
Decision Table [32]	0.89	0.11	0.89	0.79	0.96
Naïve Bayes [33]	0.87	0.14	0.87	0.74	0.94
Logistic Regression	0.88	0.12	0.88	0.76	0.91
SMO (SVM)	0.94	0.07	0.94	0.87	0.94
Random Forest	0.96	0.04	0.96	0.92	0.99

**Table 4:** Online and offline use event detection algorithm results when tested against data from the Institutional Review Board (IRB) trials.

ML Models	TPR	FPR	Precision	Recall	F-Measure	MCC	ROC Area
Online ML	0.97	0.11	0.90	0.97	0.93	0.86	0.93
Offline Rule-Based	0.92	0.23	0.80	0.92	0.86	0.70	0.85

## Appendix A. Power Consumption Calculations

*nRF51422 MCU*

Other than the time spent in a low power state, the CPU mainly consumes power when processing sensor data in a machine learning algorithm. The algorithm only runs when the cap is off. Since the majority of glaucoma patients take their eyedropper medication twice a day (Sleath 009), and from the recorded IRB trials, each instance of a patient taking their medication lasts 20 s, so the percentage of time spent in an active CPU state was calculated as follows:

(A1) $$\frac{2\;times*20\;seconds}{86,400\;seconds\;in\;a\;day}=0.046\%$$

*IMU*

There are two instances when the IMU is active and the magnetometer and accelerometer are sending data:

1. When the cap is off.
2. Every 30 min to determine if the bottle is upright to gather fluid level readings.

In the event that the bottle is not immediately upright when gathering fluid level data, the IMU continues to send data until the bottle is in an upright position or until five seconds have passed. Assuming the worst case in which the IMU continues to obtain data for five seconds every 30 min, the percentage of time spent in an active state can be calculated as follows:

(A2) $$\frac{60\;seconds\;cap\;off+\left(5\;seconds*48\;fluid\;readings\;in\;a\;day\right)}{86,400\;seconds\;in\;a\;day}=0.35\%$$

*FDC*

The capacitive sensor is active at the same instances where the IMU is active, so the percentage of time spent active is the same as that above.

*BLE*

The worst-case current consumption for BLE is found when it is in its advertising state, polling for a BLE connection. The bottle actively polls for a connection for 30 s, where it will actively advertise its information to any central device every 40 ms. This means that the bottle will poll for a connection 108,000 times in a day. When the bottle is not in its 30 s advertising interval, the bottle is instead using a counter to wait for its next interval. When advertising, the bottle sends frequent packets of data to the iPod Touch, which takes approximately 2 ms. Therefore, the percentage of time spent in an active state can be calculated as follows:

(A3) $$\frac{108,000\;times*2\;miliseconds}{86,400\;seconds\;in\;a\;day}=2.5\%$$

Given the overall daily power consumption, and the fact that the battery used to power the system is a 3.7 V 85 mAh lithium-ion battery, the life expectancy of the bottle can be calculated as follows:

(A4) $$\frac{85\;mAh*3.7V}{3.30\;mW+5.08\;mW+0.12\;mW+9.50\;mW}=17.47\;hours$$

## Appendix B. Featurization Approach

Before determining if the medication was properly dispensed, a MATLAB script first filtered the data into windows, which started when the cap was removed and ended when the cap was placed back on the bottle. In order to distinguish between properly dispensing the medication and simulating a use event, some descriptive statistics (i.e., mean, min, max, and standard deviation) were calculated using changes in the sensor data rather than the raw sensor data. The calculations are shown below:

*MIN AND MAX SLOPE:*

(A5) $$\min\left(\frac{X_{i}-X_{i-1}}{T_{i}-T_{i-1}},\;i\;\in \;\left\{1,\ldots ,n\right\}\;\right)$$

(A6) $$\;\max\left(\frac{X_{i}-X_{i-1}}{T_{i}-T_{i-1}},\;i\;\in \;\left\{1,\ldots ,n\right\}\;\right)\;$$

*SUM_ABS_SLIDE:*

(A7) $$\overset{n}{\underset{i=1}{\sum }}\frac{\left|X_{i}-X_{i-1}\right|}{T_{i}-T_{i-1}}$$

*AVG_ABS_SLIDE:*

(A8) $$\frac{\sum_{i=1}^{n}\frac{\left|X_{i}-X_{i-1}\right|}{T_{i}-T_{i-1}}}{n}$$

where *X* is the sensor data at index *i; i* is the index bounded by the start until end of window *n;* and *T* is the timestamp at index *i*.

The absolute value was included in order to make sure that the change in sensor data captured from when the bottle is tilted or squeezed is not cancelled out by the bottle expanding and moving back to its original position.

**Table A1:** Measures the percentage of total information that the model gained from each feature. The last column shows the average information gained (IG) by each sensor. The last row shows the average information gained by each algorithm. This shows that the squeeze of the bottle provides the most information to distinguish classification, followed by the orientation of the bottle.

	AVG	MIN	MAX	STDEV	MIN_SLOPE	MAX_SLOPE	SUM_ABS_SLIDE	AVG_ABS_SLIDE	Sensor IG
**FDC**	0.16	0.44	0.07	0.35	0.54	0.43	0.47	0.13	**0.32**
**ACCEL_Z**	0.47	0.00	0.33	0.31	0.21	0.23	0.32	0.31	**0.27**
**ACCEL_X**	0.21	0.17	0.00	0.18	0.12	0.19	0.22	0.17	**0.16**
**GYRO_Y**	0.00	0.12	0.13	0.19	0.00	0.08	0.22	0.16	**0.11**
**MAG_Z**	0.08	0.14	0.00	0.07	0.00	0.06	0.31	0.00	**0.08**
**GYRO_X**	0.00	0.00	0.00	0.07	0.00	0.00	0.23	0.07	**0.05**
**GYRO_Z**	0.00	0.12	0.00	0.00	0.00	0.00	0.19	0.00	**0.04**
**MAG_X**	0.00	0.00	0.07	0.00	0.00	0.00	0.19	0.00	**0.03**
**ACCEL_Y**	0.00	0.00	0.00	0.00	0.00	0.00	0.17	0.07	**0.03**
**MAG_Y**	0.00	0.00	0.00	0.00	0.00	0.00	0.10	0.09	**0.02**
**AlgorithmIG**	**0.09**	**0.10**	**0.06**	**0.12**	**0.09**	**0.10**	**0.24**	**0.10**	
